# Communication in Fungi

**DOI:** 10.1155/2012/351832

**Published:** 2011-09-26

**Authors:** Fabien Cottier, Fritz A. Mühlschlegel

**Affiliations:** ^1^School of Biosciences, University of Kent, Canterbury, Kent CT2 7NJ, UK; ^2^Singapore Immunology Network, Agency for Science, Technology, and Research, 138648, Singapore; ^3^Clinical Microbiology Service, William Harvey Hospital, East Kent Hospitals University NHS Foundation Trust, Ashford, Kent TN24 0LZ, UK

## Abstract

We will discuss fungal communication in the context of fundamental biological functions including mating, growth, morphogenesis, and the regulation of fungal virulence determinants. We will address intraspecies but also interkingdom signaling by systematically discussing the sender of the message, the molecular message, and receiver. Analyzing communication shows the close coevolution of fungi with organisms present in their environment giving insights into multispecies communication. A better understanding of the molecular mechanisms underlying microbial communication will promote our understanding of the “fungal communicome.”

## 1. Introduction

**Figure 1:**
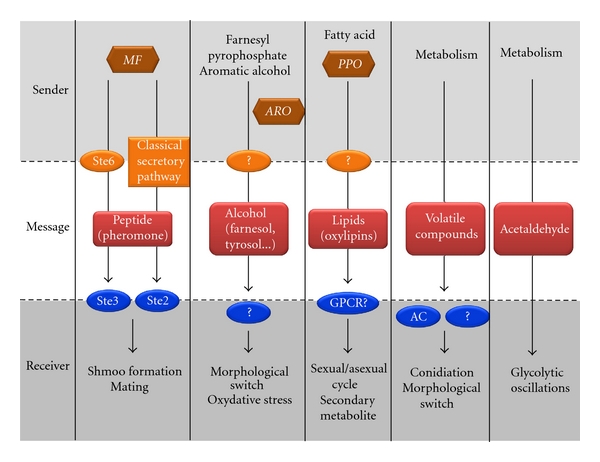
Schematic representation of fungal intra and interspecies communication. The “sender” is an organism from the fungal kingdom and the “receiver” can be from any kingdom. Genes involved in messenger synthesis are represented as brown hexagons. Proteins involved in secretion or receiving the message are in orange and blue.

Any form of communication requires the existence of three obligatory components: a sender, a message, and a receiver. The process starts with the release of a message by a sender and ends with the understanding of the message by a receiver. This type of cycle has been developed with different degrees of complexity from prokaryote to higher eukaryotes optimizing fitness and adaptation for individual members and populations. The nature and mode of action of communication is as diverse as the response to the information it carries. Inter- and intraspecies communication has been widely studied analyzing the exchange of information between fungi and bacteria or fungi and plant cells [1, 2]. This review will focus predominantly on intraspecies fungal communication addressing key biological functions including mating, growth, morphological switching, or the regulation of virulence factor expression (Figure 1). We will show that in the fungal kingdom most of these mechanisms are controlled by a variety of messengers including small peptides, alcohols, lipids, and volatile compounds.

## 2. Peptides: Pheromones

Pheromones have been known to act as an informative molecule since 1959 [3] and were reported to be involved in the sexual cycle of fungi in 1974 [4]. In the fungal kingdom, they are involved in the reconnaissance of compatible sexual partner to promote plasmogamy and karyogamy between two opposite mating types followed by meiosis. Taking the example of the extensively described sexual cycle of *Saccharomyces cerevisiae*, pheromones are diffusible peptides called a-factor (12 aa) when produced by a cells, and *α*-factor (13 aa) when produced by *α* cells. Each mating type responds to the opposite factor, and is able to produce only one of the two peptide pheromones depending on the alleles present at the *MAT* locus. Indeed, *MATa* or *MAT*α** controls the expression of a and *α* specific genes, respectively, such as genes encoding the prepro-factor and the pheromone receptor (for a comprehensive description of the *MAT* locus, see review [5]). In the example of *α* cells, *MF*α*1* encodes the pheromone precursor, prepro-*α*-factor, which undergoes several proteolytic reactions in the classical secretory pathway before releasing the mature pheromone. Contrary to the *α*-factor, the ABC transporter Ste6p is required to secrete a-factor [6]. This difference could be due to the fact that a-factors are farnesylated [7]. Once released, pheromones freely diffuse in the environment and create a concentration gradient. These peptides are subsequently recognized by a 7 transmembrane receptor present on the surface of cells: Ste2p on a cells binds the *α*-factor and Ste3p on *α* cells binds the a-factor. Ste2p and Ste3p are G-protein coupled receptors (GPCR) and the binding of pheromone induces the separation of the associated heterotrimeric G-protein into a monomeric *α* subunit GTPase (Gpa1p) and a *βγ* dimer (Ste4p–Ste18p). This mechanism results in the recruitment of Ste5p by Ste4p to the membrane, which activates a protein kinase cascade ultimately resulting in the phosphorylation of the MAP kinases Fus3p and Kss1p [8]. Once phosphorylated Fus3p migrates to the nucleus were it activates the transcriptional factor Ste12p leading to the expression of pheromone responsive genes. Phenotypically, the morphological response of cells to opposite mating pheromone is the development of a shmoo, that is, directional cell growth in response to the pheromone gradient. As each opposite cell develops a shmoo, the plasmogamy between the two cells occurs when the shmoos establish contact, starting the first step of the sexual cycle.

Contrary to *S. cerevisiae*, where karyogamy is followed by meiosis to end the sexual cycle, *Candida albicans* has not yet been described to undergo meiosis. This yeast displays only an imperfect sexual cycle, where karyogamy results in the formation of tetraploid cells that restore natural diploidy via random loss of chromosomes [9]. This process occurs only after *C. albicans* has undergone a so-called white-to-opaque switch (see review [10]), where opaque cells are the sole mating competent form of this yeast. The opaque cells morphologically respond to pheromone by producing a shmoo, like in *S. cerevisiae*, but the mating incompetent form, white cells, is also sensitive to pheromone [11]. Indeed, *C. albicansα*-factor but also a-factor promotes the formation of biofilm by white cells via enhancing their adhesiveness. A process which uses the same receptor (Ste2p or Ste3p) and transduction pathway as the response of opaque cells to pheromone [12]. The formation of fungal biomass by white cells facilitates the establishment of a pheromone gradient in a population of individual cells and assists the mating process of opaque cells. This process involves another molecule, farnesol, as the production of this molecule under aerobic conditions induces the death of the mating competent opaque cells. Anaerobic conditions that prevent production of farnesol facilitate mating between *C. albicans* opaque cells. These observations suggest that the gastrointestinal tract of humans could promote *C. albicans* mating [13]. The mechanism of pheromone communication has a broad significance in diverse fungi including ascomycetes like *Histoplasma capsulatum* [14] or *Aspergillus fumigatus* [15, 16], to basidiomycetes such as *Cryptococcus neoformans* [17] and *Ustilago maydis*, which possess a tetrapolar mating system [18]. Pheromone communication appears to be a critical mechanism for fungi as it supports the exchange of genetic material between cells and by extension the ability of the organism to evolve in response to their environment.

## 3. Alcohols: Quorum Sensing

Quorum sensing is a mechanism of communication based on the accumulation of a messenger molecule in the medium of culture [19]. As the production of messenger molecules increases with cell number, this system reflects population size. Initially discovered in bacteria, quorum sensing in fungi became relevant for the control of virulence factor expression in *C. albicans*. In 1979, Hazen and Cutler showed that the supernatant from a 48 h culture of *C. albicans* prevents the yeast to hyphae switch of a fresh culture [20]. The quorum sensing molecule (QSM) responsible for this effect has since been identified as an acyclic sesquiterpene alcohol called farnesol [21]. 

*C. albicans* produces farnesol at a rate of 0.12–0.133 mg/g of cells dry weight [22] from an intermediate of the mevalonate pathway (sterol biosynthesis), farnesyl pyrophosphate [23]. At concentrations of 10–250 *μ*M farnesol inhibits the formation of hyphae when induced with proline, N-acetylglucosamine, and serum, but does not suppress further elongation of preexisting hyphae [24]. Farnesol-dependent quorum sensing involves the histidine kinase Chk1p [25] and the Ras1-Cyr1 pathway [26] but the receptor for farnesol remains to be identified. Farnesol regulates the expression of several genes and induces *TUP1,* a transcriptional cofactor repressing filamentation [27], while repressing *CPH1* and *HST7* expression, which are both activators of the morphological switch [28]. The oxidized form of farnesol, farnesoic acid, has also been reported to inhibit hyphal growth by acting via *PHO81* [29]. However, morphological inhibition is stronger with farnesol, although farnesoic acid is less toxic at high concentration [30], it displays only 3% of farnesols QSM activity [31]. While the function of farnesol as a cell density regulator remains to be established, farnesol has been described to inhibit *C. albicans* biofilm formation due to its repressing function on the morphological switch [32]. Additionally it has been shown to increase resistance to oxidative stress by suppressing the Ras1-cAMP pathway [33].

Notably, farnesol also acts as an interspecies QSM that impacts on growth of other *Candida* species including *Candida tropicalis* or *Candida parapsilosis* [34] as well as *S. cerevisiae *or the mould *Aspergillus nidulans* and *A. fumigatus* [35–38]. In the case of *A. fumigatus*, farnesol has been described to alter the localization of AfRho1p and AfRho3p, proteins involved in the cell wall integrity (CWI) pathway and cytoskeleton regulation [35]. This phenotype is explained by the fact that farnesyl derivatives interfere with prenylated proteins such as the two Rho GTPases [39, 40]. The CWI pathway implies the activation of AfPkcA by AfRho1p, which leads on to the MAP kinase cascade and subsequent AfMpkA phosphorylation. Dichtl et al. showed that in the presence of only 40 *μ*M farnesol, phosphorylation of AfMpkA in response to Congo red was completely inhibited [35]. In *S. cerevisiae*, farnesol prevents growth via a different mechanism, which involves an increase of mitochondrial reactive oxygen species (ROS) [37]. The latter observation was also reported for *A. nidulans* where ROS augmentation induced cellular apoptosis but had no role on hyphal morphogenesis [38]. Two proteins have been identified in this response; the G*α* subunit FadA of a heterotrimeric G protein, where hyperactivation leads to a strong increase in farnesol sensitivity [38], and the kinase PkcA. Mutation of PkcA increases resistance to farnesol while overexpression results in a higher rate of cell death in response to the QSM [41]. Finally, farnesol has also been described to induce apoptosis of cancerous cells *in vivo* (see review [42]), as well as increasing antibiotic sensitivity of *Staphylococcus aureus* [43]. Thus, farnesol appears to function as both an intraspecies and inter-species communication molecule.

Farnesol is not the only continuously released messenger molecule by *C. albicans*. Tyrosol, an aromatic alcohol, is produced from aromatic amino acids undergoing the processes of transamination (*ARO8, ARO9*), decarboxylation (*ARO10*), and reduction by alcohol dehydrogenase (*ADH*) [44]. This synthesis pathway is strongly dependant on growth conditions including environmental pH, availability of aromatic amino acids, oxygen levels, or presence of ammonium salts [44]. Similar to farnesol, tyrosol's sensor has not yet been identified. Fungal responses to tyrosol include the induction of germ tubes in planktonically growing cells and during the early stages of biofilm formation, as well as a reduction in the lag phase of *C. albicans* growth following dilution of a highly concentrated culture to fresh minimal medium [45, 46]. The latter phenotype occurs predominantly at low concentrations of cells (5 × 10^3^ cell/mL) by promoting the expression of genes involved in DNA replication, chromosome segregation, and cell cycle processes [45].

Aromatic alcohol synthesis is not exclusive to *C. albicans* but can also be found in *S. cerevisiae*, which produces phenylethanol and tryptophol via a similar pathway involving AROs genes [47]. Both molecules stimulate diploid pseudohyphal growth at concentrations above 20 *μ*M on low-ammonium agar (SLAD) by inducing the PKA pathway resulting in *FLO11* induction [48]. Recently, response to phenylethanol and tryptophol has been proposed to involve two main transcriptional regulators: Cat8p and Mig1p [49]. Interestingly, *C. albicans* is insensitive to phenylethanol and tryptophol [48]. *H. capsulatum* and *Ceratocystis ulmi *are two fungi also displaying quorum sensing phenotypes. However, the messenger molecule is not yet characterized [50, 51]. At low density, *H. capsulatum* cells have low amounts of *α*-(1,3)-glucan in their cell walls and addition of supernatant from a stationary phase culture induces *α*-(1,3)-glucan incorporation into the cell wall [50]. Similarly addition of *C. ulmi* spent medium to a fresh culture promotes a switch from hyphae to yeast growth [51].

## 4. Lipids: Oxylipins

Oxylipins are oxygenated fatty acids used as cell messengers and have been intensely studied in plants and mammalian cells (see review [52]). They also appear to be widely synthesized and secreted by fungi.* A. nidulans* was reported to produce one of the first oxylipins called psi factor (precocious sexual inducer), which is composed of a series of different oxylipin derivates from oleic acid (C18:1), linoleic acid (C18:2), and linolenic acid (C18:3) called psiA, psiB, and psiC, respectively [53]. The genes involved in the production of psi factor are called *Ppo*s (for psi-producing oxygenases) [54]. In the case of *A. nidulans*, *PpoA *is involved in psiB*α* synthesis and *PpoB* and *PpoC* contribute to psiB*β* biogenesis [54, 55]. Inactivation of these genes results in perturbations not only of psi factor production but also mycotoxins production, as well as in the ratio between the development of sexual and asexual ascospores [54, 55]. The latter phenotype is due to the fact that oxylipins control the expression of *NsdD* and *BrlA*, transcription factors required for meiotic and mitotic sporulation, respectively [54, 56]. Overexpression or addition of psiB*α* or psiC*α* to the culture medium stimulates sexual sporulation and represses asexual spore development while an opposite effect is observed for psiA*α* and psiB*β* [55]. Secondary metabolite mycotoxin sterigmatocystin (ST) and antibiotic penicillin (PN) production are also dependent on oxylipin [57]. Indeed, inactivation of the *Ppo*s genes results in the inability to secrete ST as a result of downregulation of ST biosynthesis genes including *aflR *and *stcU*, and the overproduction of PN through induction of the gene involved in its biosynthesis: *ipnA* [57]. Interestingly, the exact opposite observations are found when FadA, an *α* subunit of a GPCR, is constitutively activated due to a G42R mutation, a reaction which is mediated by the PkaA enzyme [58]. This result, in addition to the fact that oxylipins in mammalian cells are sensed via GPCR complexes [59] led to the hypothesis that fungi use the same system to detect oxylipin, ultimately activating the cAMP/PKA pathway [58]. The *ppo* and GPCR encoding genes have been identified in the genomes of several filamentous fungi predicting a broader role of oxylipin in fungal biology [60]. In fact, inactivation of the *ppo* genes in *Aspergillus flavus* and *Fusarium sporotrichioides* has already been shown to perturb mycotoxin and spore production [61, 62]. Finally, using confocal laser scanning microscopy, oxylipins have been described to accumulate in the capsule of *C. neoformans* before being released into the external medium under the form of hydrophobic droplets that are transported via tubular protuberances [63].

Recently, Nigam et al. have described a 3(*R*)-Hydroxy-tetradecanoic acid, a derivate of linoleic acid as a novel QSM of *C. albicans* [64]. Previously known to be produced during the sexual phase of *Dipodascopsis uninucleata* [65], this oxylipin increases filamentation in *C. albicans* in response to N-acetylglucosamine at a concentration of 1 *μ*M. Although the receptor of 3(*R*)-Hydroxy-tetradecanoic is not known, this QSM induces *HWP1* and *CAP1* mRNA transcripts [64]. Interestingly, 3(*R*)-Hydroxy-tetradecanoic is metabolized inside cells to generate two new compounds that could also act as messenger molecules [64].

Another family of oxylipin are the eicosanoids, which are molecules containing a 20 carbon backbone [66]. PGE_2_ is produced by *C. albicans* from exogenous arachidonic acid via enzymes not yet characterized [67]. PGE_2_ is also produced by humans, similar to other prostaglandins (PG), and it appears that fungal PGE_2_ can enter in competition with human PG impacting on the host's immune response [67]. Indeed, PGE_2_ is known to balance Th1/Th2 differentiation as this molecule decreases the expression of IL-12R and inactivates Th1 differentiation while activating the Th2-related immune responses [68]. PGE_2_ also enhances the production of IgE in stimulated B cells [69].

## 5. Volatile Compounds and Gas

In addition to releasing mediators into solution or onto solid growth media, organisms also exchange information via the liberation of messenger molecules into air. For example, insects have been thoroughly studied for their secretion of pheromones into air to attract mating partners [70]. In the fungal kingdom, as early as in the 1970s, volatile compounds from fungi and others organism have been described to impact on fungal growth (review [71, 72]). More recently, Palkova et al. observed that *S. cerevisiae* colonies grown on complex agar form a turbid path in the vicinity of another colony. Subsequently, they discovered that this reaction is induced by the small volatile messenger molecule, later described as ammonia [73], which also required amino-acid uptake for its production. Indeed, inactivation of *SHR3*, which is responsible for the correct localization of several amino-acid permeases, disrupts the turbid path between colonies [73]. 

*Trichoderma* species have been described to produce the volatile molecule 6-Pentyl-*α*-pyrone, a secondary metabolite with antifungal activity [74]. However, more recently the induction of conidiation in *Trichoderma* species, which is known to be regulated by a circadian cycle, has also been shown to be controlled via a volatile agent. Solid-phase microextraction linked with gas chromatography and mass spectrometry has allowed the identification of the chemical profiles of volatile molecules produced from nonconidiated colonies grown in darkness and conidiating colonies grown in light [75]. Comparison of the two profiles identified production of the 8-carbon compounds molecules 1-octen-3-ol, 3-octanol and 3-octanone specifically during conidiation [75]. Each of these three compounds induces conidiation in colonies placed in the dark. This regulation could involve a calcium-dependant signaling pathway as it has been shown that high concentration of calcium can induce conidiation of *Penicillium isariaeform* in darkness [76]. 1-octen-3-ol is the most efficient molecule being active at concentrations of only 0.1 *μ*M. Interestingly, concentrations above 500 *μ*M of any of the three compounds suppress conidiation and growth of *Trichoderma* species. These observations are consistent with a previously described putative fungistatic and fungicidal role of the molecules [77, 78]. Notably, the same compounds have previously been shown to function as insect attractants improving fungal spore dispersal [77, 78], and inter-species communication has already been described between *Epichloë* species and the female *Botanophila* flies [79].

Fungi are not only responsive to volatile compounds that they produce but also, in at least one example, to a gas liberated during respiration: carbon dioxide (CO_2_). As early as 1961, Vakil et al. demonstrated that the optimum CO_2_ concentration for the germination of *Aspergillus niger* conidiospores is reached not under normal atmospheric concentrations of CO_2_ (0.033%) but at 0.5% [80]. Since then several additional phenotypes in fungi have been attributed to changes in the concentration of environmental CO_2_ including the sporulation of *Alternaria crassa* and *Alternaria cassiae *[81], conidiation of *Neurospora crassa* [82], or capsule formation and mating in *C. neoformans* [83, 84]. 

Recently, significant advances have been made in the understanding of CO_2_ sensing in fungi. It was already known that the yeast to hyphae morphological switch in *C. albicans* is triggered by elevated environmental CO_2_ [85]. Furthermore, the frequency of white-to-opaque switching can be increased 16-fold in hypercapnic conditions as opposed to atmospheric CO_2_ [86]. Two different studies show that both phenotypes involve the *C. albicans* adenylyl cyclase Cyr1, first fungal CO_2_ sensor. This enzyme generates the secondary messenger cAMP, which in the context of the cAMP/PKA signaling pathway has a fundamental impact on *C. albicans* morphogenesis [86, 87]. CO_2_ activation of Cyr1p depends on the concentration of bicarbonate, the hydrated form of CO_2_ [87]. CO_2_ hydration occurs naturally at a very low rate, but is enhanced by the enzyme carbonic anhydrase [88]. Inactivation of *CYR1* results in a loss of filamentation and white to opaque switching frequency in response to hypercapnia [86, 87]. Hall et al. have now demonstrated that Lysine 1373 of the Cyr1 catalytic domain is essential for CO_2_ sensing in *C. albicans* as mutation of this amino acid leads to a loss of filamentation in response to CO_2_ but not to serum, another morphological cue [89]. These data show that in fungi environmental CO_2_ is sensed via the adenylyl cyclase, which transduces the message via the regulation of the cAMP/PKA pathway. Hall et al. also showed that hypercapnia is not a condition solely encountered inside the host but can also establish itself as a population event, such as the center of a colony grown under normal atmospheric conditions [89]. Another study demonstrated that *C. albicans* produces CO_2_ via the conversion of arginine to urea. Urea is ultimately degraded to generate CO_2_ by the enzyme urea amidolyase (Dur1,2). Inactivation of the latter interferes with *C. albicans* filamentation in response to arginine and urea compared to the control strain but not to elevated CO_2_ [90].

Control of the white to opaque switch-frequency in *C. albicans* by environmental CO_2_ also involves the GTPAse Ras1 and the transcriptional factor Wor1. Indeed, Ras1, Cyr1, and Wor1 are critical for increasing the white to opaque switch in response to concentrations of CO_2_ at 1%, but Ras1 and Cyr1 become optional for the induction at higher concentrations (20%). However, Wor1 remains essential for the switch even at high CO_2_ [86]. These results imply that an alternative CO_2_ sensing pathway is involved in the regulation of Wor1 at high CO_2_ in *C. albicans*. However, it is important to note that under this condition a significant increase of the internal pH may occur which could also be a component of this alternative CO_2_ sensing pathway.

## 6. Small Molecule: Acetaldehyde

Acetaldehyde, an organic compound involved in several cellular pathways, has been shown to impact on cell-density-dependent glycolytic oscillations of *S. cerevisiae* [91]. In 1964, Chance et al. described that the level of NADH in yeast oscillated when starved cells endure a pulse of glucose after a switch to anaerobic conditions [92]. Since then other metabolites have been described to oscillate in yeast including glucose-6-phosphate, fructose-6-phosphate, fructose-1,6-biphosphate, AMP, ADP, and ATP (for a comprehensive review see [93]). Interestingly, at a population level these oscillations are not chaotic but appear to be subject to synchronization. The most striking observation was achieved when mixing two populations with a 180° out-of-phase oscillation showing that within minutes the oscillation of the new population were synchronized [91]. Acetaldehyde was identified as the active molecule in the synchronization of these oscillations, as the use of acetaldehyde traps induced the oscillation to be damped and addition of acetaldehyde to the medium produced a phase shift in the oscillation [91]. Acetaldehyde is a small molecule that can passively diffuse through the cell membrane. No specific target for acetaldehyde is known; however, this compound has an important impact on the NAD^+^/NADH balance [94]. 

Acetaldehyde is also a volatile molecule, a property used to study inter and intraspecies communication in a synthetic ecosystem [95]. By engineering sender cells that liberate volatile acetaldehyde and receiver cells that contain a construct under an acetaldehyde-inducible promoter, it was possible to study volatile cell communication in a controlled environment. Using mammalian (CHO-K1), bacterial (*Escherichia coli*), yeast (*S. cerevisiae*), or plant (*Lepidium sativum*) cells, all combination of sender/receiver for inter and intraspecies resulted in a positive communication between cells [95]. These results show that virtually all cells can communicate with themselves or different species. Clearly such models could bring new insight in the understanding of communication in complex living systems.

## 7. Concluding Remarks and Outlooks

We are currently at an interesting stage in the understanding of fungal communication. Many essential compounds of the communication process have been identified, the sender (in our case fungi), the message (protein, alcohol, lipid, gas), and the receiver (bacteria, fungi, plant, mammalian). However, in most cases the actual molecular mechanism of such communication remains for most parts unknown. The determination of these pathways is of substantial significance as molecular messengers control the expression of fungal virulence determinants including the yeast-to-hyphae switch and biofilm formation in* C. albicans*, capsule formation in *C. neoformans*, or mycotoxin synthesis in *A. nidulans*, but also the propagation of these organisms via the regulation of their sexual and asexual cycle. A better knowledge of fungal communication is now required to permit the development of innovative strategies aiming to control disease or toxin production of these organisms. 

Fungi have already taken advantage of the different communication processes and particularly inter-species communication to gain competitive advantages over other species. Good examples are the production of pollinators attracting insects to give phytopathogenic fungi a better chance for dispersal of their spores [79]. Additionally, synthesis of PGE_2_ by the human pathogens *C. albicans* and *C. neoformans* modify the host immune response and may enhance fungal survival [67]. Such mechanisms reveal the close coevolution of fungi with their environmental partner and give insights into multispecies communication. The remarkable versatility of communication in the fungal kingdom also raises the question how these organisms integrate intra- and inter-species messages that can have opposing effects. As the molecular mechanisms of fungal communication unravel further, they will promote our understanding of the highly attractive but challenging topic of the fungal “communicome.”
